# Clinical outcome and quality of life in octogenarian patients with muscle-invasive urothelial carcinoma of the bladder treated with radical cystectomy or transurethral resection of the bladder tumor: a retrospective analysis of 143 patients

**DOI:** 10.1007/s11255-021-03073-5

**Published:** 2021-11-24

**Authors:** Christian Rehme, Beatrix Fritsch, Luca Thomas, Stefan Istin, Carolin Burchert, Bastian Hummel, Bogdan Baleanu-Curaj, Henning Reis, Tibor Szarvas, Herbert Ruebben, Boris Hadaschik, Christian Niedworok

**Affiliations:** 1 Department of Urology, University Hospital Essen, University of Duisburg-Essen, Hufelandstr. 52, 45147 Essen, Germany; 2 Department of Urology, Hermann-Josef-Hospital, Erkelenz, Germany; 3 Institute of Pathology, University Hospital Essen, University of Duisburg-Essen, Hufelandstr. 52, 45147 Essen, Germany; 4 Department of Urology, Semmelweis University, Budapest, Hungary; 5 Department of Urology, Helios Hospital Duisburg, Duisburg, Germany

**Keywords:** Muscle-invasive bladder cancer, Transurethral resection, Octogenarian, Cystectomy, Urothelial carcinoma

## Abstract

**Purpose:**

To compare clinical outcome and quality of life (QoL) in octogenarian patients with muscle-invasive urothelial carcinoma (MIBC) either treated by radical cystectomy (RC) or transurethral resection of the tumor (TURBT).

**Methods:**

We identified octogenarian patients with MIBC in our institutions since 2005. Clinical treatment outcomes and QoL were analyzed. Uni- and multivariable Cox regression analyses, two-tailed Wilcoxon test, Mann–Whitney test and Fisher’s exact test were assessed as appropriate. QoL was evaluated using FACT-G (Functional Assessment of Cancer Therapy-General) questionnaire.

**Results:**

143 patients were identified (RC: 51 cases, TURBT: 92 cases). Mean follow-up was 14 months (0–100 months). Median overall survival (OS) was 12 months in the RC group and 7 months in the TURBT group. TURBT and low preoperative hemoglobin were independent risk factors for reduced cancer-specific survival (CSS) (TURBT: *p* = *0.019*, Hb: *p* = *0.008*) and OS (TURBT: *p* = *0.026*, Hb: *p* = *0.013*) in multivariable analyses. Baseline QoL was low throughout the whole cohort. There was no difference in baseline FACT-G scoring comparing RC and TURBT (FACT-G total score (median): RC 43.7/108 vs. TURBT 44.0/108, *p* = *0.7144)*. Increased FACT-G questionnaire scoring was assessed for RC patients (median percentage score change RC 22.9%, TURBT 2.3%, *p* < *0.0001*).

**Conclusion:**

RC and TURBT are feasible treatment options for MIBC in octogenarian patients. In our cohort, RC was associated with increased CSS, OS and QoL. QoL in general was low throughout the whole cohort. Interdisciplinary decision-making has to be improved for these critically ill patients.

## Introduction

Since the 1950s, average worldwide life expectancy increased by 22.4 years. Babies born today have a life expectancy of 73.5 years. In countries with a high socio-demographic index, life expectancies are even higher, reaching 81.2 years [1]. Although cardiovascular disease still is the leading cause of death, incidences of many malignancies continuously increase [1]. Bladder cancer (BC) is the second-most common malignancy in urology with a widespread incidence of 430,000 cases worldwide. In 75%, the patients are male. Eighty percent of urothelial BCs are non-muscle-invasive at first diagnosis while another 20% are infiltrating the muscular layer of the bladder [2]. Muscle-invasive bladder cancer (MIBC) is characterized by a reduced prognosis with a 5-year cancer-specific survival (CSS) of 67%, justifying radical multidisciplinary treatment [3]. The standard treatment of MIBC is radical cystectomy (RC) with pelvic lymph node dissection [4]. In the treatment of oncological diseases, preserving patients` quality of life plays a pivotal role. Especially in geriatric patients, a well-balanced decision should be made between cancer treatment with its consecutive side effects and the option of best supportive care or non-radical treatment [5]. There are different treatment options for MIBC in geriatric patients. Taking into account the general physical condition, patients at an advanced age may be treated surgically by RC, by transurethral resection of the tumor (TURBT) or by combined radio-chemotherapy, at present. Surgical and anaesthesiological techniques have improved during the last decades increasingly enabling radical surgery in octogenarians.

The aim of the present study was to evaluate clinical outcome and quality of life (QoL) after RC or TURBT for MIBC in octogenarian patients.

## Patients and methods

From our database, we identified 9176 patients with newly diagnosed urothelial carcinoma which were treated at the Urology Departments at University Hospital Essen and Hermann-Josef Hospital Erkelenz between 2005 and 2021. Initial TURBT treatment was performed in all patients to gain tumor tissue samples and to perform local tumor staging. MIBC was identified in 1176 cases of which 147 were at an age ≥ 80 years. In all patients with muscle-invasive disease, computed tomography (CT) scan of chest and abdomen was performed. Inclusion criteria for the present study were presence of MIBC of the lower urinary tract, patient`s age ≥ 80 years at the time of the diagnosis, adequate clinical staging (CT scan of chest and abdomen) and a written informed consent. Exclusion criteria were inadequate clinical staging, simultaneous urothelial carcinoma of the upper urinary tract and disagreement to written informed consent. 143 patients met the full inclusion criteria and were included in the study protocol. All subjects were followed up from baseline (date of diagnosis of MIBC) to June 2021.

### Techniques

Decision on tumor-related therapy was made after presentation of the case at the institutional multidisciplinary tumor board. In accordance with the patients` preferences, the decision for the appropriate surgical technique was done after geriatric assessment. None of the patients received neoadjuvant or adjuvant chemotherapy. When TURBT was chosen as definitive treatment option, the tumor was radically resected. In asymptomatic patients, TURBT was repeated annually. If required, additional TURBT was performed when a patient was suffering from acute or chronic bleeding or other symptoms due to local tumor burden (e.g. tumor-related irritative urinary incontinence or obstructive voiding disorder). RC as treatment option was performed as open surgery with radical resection of the bladder and the regional pelvic lymph nodes. An ileal conduit or ureterocutaneostomy was performed as urinary diversion.

### Quality of life (QoL)

Since 2014, QoL was assessed prospectively using the Functional Assessment of Chronic Illness Therapy Measurement System (FACIT) of health-related quality of life [6]. The patients were asked to answer to its 27 items Functional Assessment of Cancer Therapy-General (FACT-G, version 4), to focus on cancer-related symptoms. The FACT-G questionnaire includes 4 modules (physical, social/family, emotional and functional well-being). License was obtained from the license holder by the corresponding author. Score range is 0–108. The higher the score, the better the quality of life. A baseline score was determined at the time of diagnosis of MIBC. 6 months after tumor therapy (RC vs. TURBT), a follow-up survey was performed.

### Follow-up and evaluation of complications and comorbidities

Clinical and pathological data were retrospectively obtained from the patient`s charts and medical reports in the outpatient`s department and from the treating office-based urologists. Last routine follow-up was June 2021. Postoperative complications were evaluated according to the classification of Clavien and Dindo [7]. The highest reached Clavien–Dindo classification score during the individual resections was taken as applicable value. Patients’ comorbidity status was assessed using the Charlson comorbidity index (CCI) [8]. Multi-medication was defined as administration of four or more different preparations.

### Statistical analysis

Data are presented as medians ± standard error of the mean (SEM). Statistical significance was assigned at the level of *p* < 0.05. Data lacking normal distribution were analyzed by the non-parametric two-tailed Wilcoxon matched-pairs signed-rank test for paired group comparisons or the Mann–Whitney test for independent data as appropriate. Proportional distribution of the clinicopathological results was analyzed using the Fisher’s exact test. Overall survival (OS) analyses were done by uni- and multivariable Cox proportional hazard survival regression analyses and Kaplan–Meier survival analyses with log-rank (Mantel–Cox) test, using IBM® SPSS® (version 24.0, Chicago, IL, USA) and GraphPad Prism® (version 9, La Jolla, CA, USA). Primary endpoints of the present study were OS and CSS. Secondary endpoints were QoL and change in QoL.

## Results

### Study population

Median patient`s age was 83 years (range: 80–99 years) (Table 1). The male to female ratio was 2:1. Endoscopically treated patients were older than the patients who received RC (84.5 years vs. 82.0 years, *p* < *0.0001*). There was no difference in preoperative hemoglobin, glomerular filtration rate (GFR) and body mass index between the two groups. In general, serum hemoglobin and GFR were reduced in both groups (median hemoglobin 11.7 g/dl (reference > 12 g/dl) and median GFR 53.7 ml/min (reference > 90 ml/min)), reflecting the fact that the study cohort consists of geriatric patients. There was no difference in tumor grading, CCI and Clavien–Dindo classification score in both groups. Complications ≥ grade 3 in Clavien–Dindo classification were seen in one-third of the patients (Table 2). CCI was ≥ 6 scores in 42.7% of the cohort (Table 2).

**Table 1:** Age, hemoglobin, glomerular filtration rate and body mass index (BMI) as determined at the time of diagnosis of MIBC

	*n*	All		*n*	RC		*n*	TURBT		*p*
		Median	Range		Median	Range		Median	Range	
Age (Years)	143	83.0	80–99	51	82.0	80–89	92	84.5	80–99	< 0.0001
Hemoglobin (g/dl)	143	11.7	6.0–18.2	51	12.0	6.0–17.2	92	11.6	7.8–18.2	0.6660
Glomerular filtration rate (ml/min)	143	53.7	6–131	51	61	16–131	92	46	6–96	0.0822
BMI (kg/m^2^)	135	23.3	17.0–42.4	50	23.0	18.3–37.6	85	23.3	17.0–42.4	0.6587

**Table 2:** Patient characteristics in the whole cohort and in the subgroups of patients treated by RC and TURBT

		All		RC		TURBT		*p*
		*n*	%	*n*	%	*n*	%	
T	T2	113	79.0	37	72.5	76	82.6	0.1805
	T3	16	11.2	9	17.6	7	7.6	
	T4	14	9.8	5	9.8	9	9.8	
N/M	Negative	101	70.6	42	82.4	78	84.8	0.1755
	Positive	42	29.4	9	17.6	14	15.2	
G	2	27	18.9	6	11.8	21	22.8	0.1226
	3	116	81.1	45	88.2	71	77.2	
ASA-PS	1	1	0.7	1	2.0	–	–	0.0425
	2	39	27.3	20	39.2	19	20.7	
	3	68	47.6	23	45.1	45	48.9	
	4	23	16.1	5	9.8	18	19.6	
	5	12	8.4	2	3.9	10	10.9	
Charlson Comorbidity	Scores 1*–*5	82	57.3	27	52.9	55	59.8	0.4819
Index	Scores ≥ 6	61	42.7	24	47.1	37	40.2	
Complications (Clavien–Dindo Classification)	Grade 0	7	4.9	3	5.9	4	4.3	0.8557
	Grade 1	32	22.4	13	25.5	19	20.7	
	Grade 2	59	41.3	20	39.2	39	42.4	
	Grade 3	29	20.3	8	15.7	21	22.8	
	Grade 4	4	2.8	2	3.9	2	2.2	
	Grade 5	12	8.4	5	9.8	7	7.6	
Multi-medication	1–3 preparations	45	31.5	17	33.3	28	30.4	0.7121
	≥ 4 preparations	98	68.5	34	66.7	64	69.6	

*ASA-PS* American Society of Anesthesiologists physical status classification, *BMI* body- mass index, *G* differentiation of tumor tissue, *M* metastases, *N* lymph nodes, *T* local tumor staging)

As histopathologically evaluated local tumor extend was only available for the RC group we decided to compare the clinical CT scan-based tumor staging in both groups. Otherwise, underestimating of local tumor extend in the TURBT might be expected. There was no difference in preoperatively performed local tumor staging, status of lymph nodes and visceral metastases assessed by CT scan in both groups. Preoperative imaging-based clinical staging showed T2: 72.5%, T3: 17.6% and T4: 9.8% for the RC group compared to T2: 82.6%, T3: 7.6% and T4: 9.8% in the TURBT group (*p* = *0.1805*, *n* = 143). After performing RC, in approximately half of the cases, a higher local tumor status, positive lymph nodes or visceral metastases were identified by histopathological examination compared to preoperative CT scan results. There was no crossover of patients from the TURBT group into the RC group. American Society of Anesthesiologists physical status (ASA-PS) classification was reduced in the TURBT group compared to the RC group (*p* = *0.0452*). ASA-PS 4 and 5, representing severe physical limitations were found more often in the TURBT group (ASA-PS 4 and 5 in 19.6% and 10.9%) compared to the RC group (9.8 and 3.9%) (Tables 1 and 2).

Multi-medication (defined as intake of ≥ 4 preparations daily) was seen in almost two third of the patients in both groups (66.7% in the RC and 69.6% in the TURBT group) (Tables 1 and 2).

### Survival

**Fig. 1:**
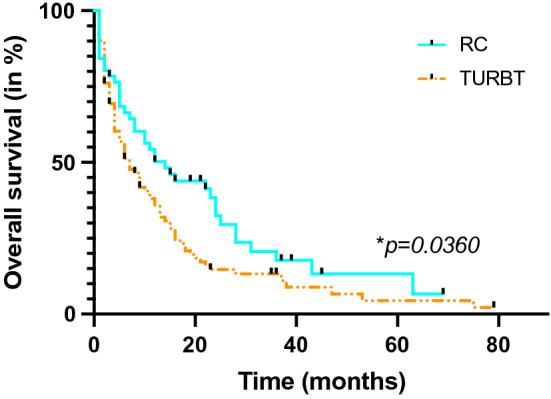
Kaplan–Meier survival curves for OS stratified by treatment method (RC or TURBT)

Mean follow-up was 14 months (0–100 months). Median OS was 12 months in the RC group and 7 months in the TURBT group. There were 80 deaths in the TURBT group and 39 deaths in the RC group. 1, 2 and 5 years OS was 42.7, 25.9 and 18.9% for the whole cohort. Stratified according treatment method, OS was 39.1, 20.7 and 15.2% in the TURBT group and 49.0, 37.2 and 25.5% in the RC group. In-house mortality and mortality within 30 days after surgery was 7.0% for the whole cohort (4.3% in the TURBT group (4/92 cases) and 11.8% in the RC group (6/51 cases; *p* = *0.1671*)). Kaplan–Meier survival probability test showed a reduced OS for TURBT patients compared to RC patients in the present cohort (*p* = *0.0360*) (Fig. 1).

Univariable Cox regression analyses identified high ASA-PS, TURBT and low preoperative hemoglobin as significant risk factors for reduced OS and CSS (ASA-PS: OS *p* = *0.044*, CSS *p* = *0.043*; treatment method: OS *p* = *0.005*, CSS *p* = *0.006* and hemoglobin: OS *p* = *0.006*, CSS *p* = *0.003*). In addition, higher Clavien–Dindo classification score was identified as risk factor for reduced OS (*p* = *0.030*) (Table 3).

**Table 3:** Univariable Cox regression analyses for OS and CSS

	*n*	Cancer specific survival			Overall survival		
		HR	95% CI	*p *value	HR	95% CI	*p *value
Age (≤ 83 vs. > 83 years)	143	1.625	0.987–2.673	0.051	1.441	0.986—2.105	0.059
Sex (male vs. female)	143	0.920	0.550–1.538	0.750	0.987	0.667—1.459	0.947
ASA-PS (≤ 2 vs. ≥ 3)	143	1.831	1.020–3.290	0.043	1.561	1.013—2.407	0.044
Treatment (RC vs. TURBT)	143	1.460	1.115–1.910	0.006	1.336	1.093—1.633	0.005
Hemoglobin (normal vs. anemia)	143	0.370	0.193–0.709	0.003	0.536	0.342—0.839	0.006
Glomerular filtration rate (normal vs. insufficient)	143	0.770	0.242–2.452	0.658	0.598	0.220—1.622	0.312
BMI (normal vs. over-/underweight)	135	0.752	0.445–1.271	0.287	0.856	0.577–1.270	0.440
T (organ confined T2 vs. locally advanced T3/T4)	143	1.112	0.686–1.803	0.666	1.030	0.707–1.501	0.878
N/M (negative vs. positive)	143	1.125	0.921–1.373	0.248	1.058	0.863–1.296	0.587
Grading (G2 vs. G3)	143	0.511	0.261–0.999	0.050	0.723	0.454–1.151	0.172
Multi-medication (< 3 preparations vs. ≥ 3 preparations)	143	0.810	0.484–1.356	0.423	0.830	0.559–1.232	0.356
Clavien–Dindo classification score (I–II vs. ≥ IIIa)	141	1.113	0.658–1.882	0.690	1.538	1.043–2.268	0.030
Charlson comorbidity index (1–5 vs. ≥ 6)	138	1.115	0.692–1.797	0.654	1.010	0.696–1.465	0.960
FACT-G scoring (≤ 43.8 vs. > 43.8)	69	0.789	0.434–1.433	0.437	0.826	0.420–1.626	0.580

In the multivariable analyses, TURBT and low preoperative hemoglobin were shown to be independent risk factors for reduced OS and CSS (treatment: OS *p* = *0.026* and CSS *p* = *0.019*; hemoglobin: OS *p* = *0.013* and CSS *p* = *0.008*) (Table 4).

**Table 4:** Multivariable Cox regression analyses for OS and CSS

	*n*	Cancer specific survival			Overall survival		
		HR	95% CI	*p *value	HR	95% CI	*p *value
ASA-PS (≤ 2 vs. ≥ 3)	143	1.507	0.827–2.746	0.180	1.283	0.806–2.043	0.293
Treatment (RC vs. TURBT)	143	1.387	1.057–1.822	0.019	1.262	1.028–1.548	0.026
Hemoglobin (normal vs. anemia)	143	0.410	0.212–0.791	0.008	0.560	0.354–0.887	0.013
Clavien–Dindo classification score (I–II vs. ≥ IIIa)	141	–	–	–	1.432	0.953–2.152	0.084

### Quality of life

Pre- and postoperative health-related QoL questionnaires were obtained from all 69 patients (RC *n* = 26, TURBT *n* = 43) who were treated since 2014. In the earlier years, no QoL assessment was performed. Baseline total FACT-G score was low (summary: 43.8/108) throughout the whole cohort. Baseline scores showed no difference comparing RC and TURBT patients (FACT-G total score (median): RC 43.7/108 vs. TURBT 44.0/108, *p* = *0.7144*; subscales: physical well-being (PWB): RC 10.0, TURBT 10.0, *p* = *0.7083*; social/family well-being (SWB): RC 13.5, TURBT 12.8, *p* = *0.1497*; emotional well-being (EWB): RC 10.0, TURBT 10.0, *p* = *0.4587* and functional well-being (FWB): RC 10.0, TURBT 10.0, *p* = *0.5433*). Maximum score achievable was 28 for PWB, SWB and FWB and 24 for EWB.

**Fig. 2:**
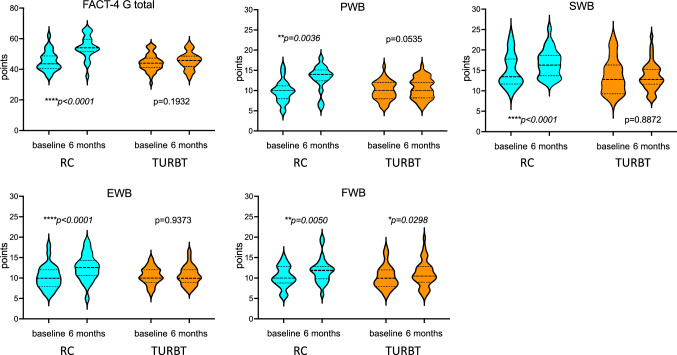
Health-related QoL. *PWB* physical well-being, *SWB* social/family well-being, *EWB* emotional well-being, *FWB* functional well-being, administered by the FACT-G (version 4) questionnaire (*n* = 69)

Following surgery, 6-month follow-up QoL scoring was increased in all subscales for RC (median score changes: FACT-G total + 10.3, *p* < *0.0001*, PWB + 13.5, *p* = *0.0036*; SWB + 16.3, *p* < *0.0001*; EWB + 12.5, *p* < *0.0001* and FWB + 11.85, *p* = *0.0050*) and FWB in the TURBT group (+ 10.5, *p* = *0.0298*). There was no change in TURBT for FACT-G total (+ 1.0, *p* = *0.1932*) and PWB (+ 10.0, *p* = *0.0535*), SWB (+ 12.8, *p* = *0.8872*) and EWB (+ 10.0, *p* = *0.9373*) (Fig. 2). When comparing the median percentage of change from the baseline scores, we found improvement in the PWB and EWB subgroup and the FACT-G score for RC patients (PWB: RC 40%, TURBT 0%, *p* < *0.0001*; EWB: RC 22.8%, TURBT 0%, *p* = *0.0001*; FACT-G: RC 22.9%, TURBT 2.3%, *p* < *0.0001*).

**Fig. 3:**
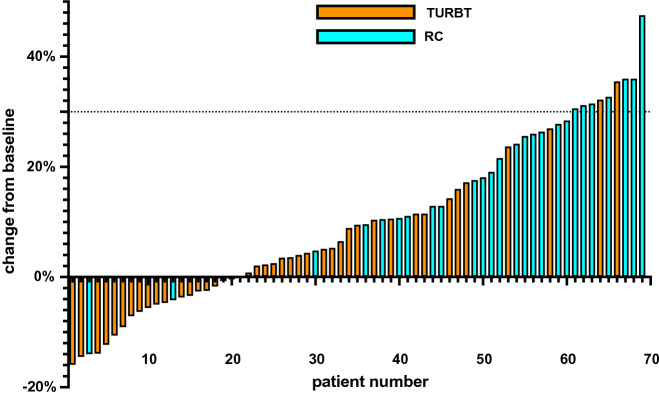
The waterfall plot of percentage of score change in health-related QoL

QoL improvement was predominant in the RC group. In 9/69 cases, a change of more than 30% from baseline was observed. In seven of these cases, RC was performed, TURBT was done in two of these cases (Fig. 3).

## Discussion

In the present study, we show CSS, OS and QoL are increased for RC compared to TURBT in a cohort of octogenarian patients with MIBC.

As shown in several studies, RC is a veritable treatment option for MIBC in elderly patients [9–16]. Some results, indeed, point out a benefit for RC as compared to bladder-sparing with TURBT in elderly patients even with multiple comorbidities and at a high risk for perioperative morbidity [17–19]. However, controversy remains since advanced age is an independent risk factor for reduced CSS in patients treated with RC for MIBC [12, 20]. The influence of mental health on BC survival remains unclear [21, 22]. Nevertheless, an under-treatment of elderly patients needs to be avoided wherever possible [23]. An important limitation for radical treatment of MIBC is poor patient`s physical condition. In the present study, although representing a retrospective investigation, the patient cohort is quite homogenous concerning comorbidities and multi-medication. On the other hand, ASA-PS was worse in the TURBT group, reflecting the fact that especially in geriatric patients, preoperative physical condition is a basic prerequisite for radical surgery.

In all 51 cases treated by surgery, RC with incontinent urinary diversion (ileal conduit or ureterocutaneostomy) was performed. We critically reflect these results, since it was shown in another cohort that at least some of the elderly patients can be quite suitable for orthotopic urinary diversions [24]. Sogni et al. showed that severe complications were uncommon and QoL in the patients with orthotopic urinary diversion was comparable to the patients with incontinent diversions. On the other hand, it is worth mentioning, that incontinence in the subgroup of patients > 75 years was very common (75% nighttime and 46% daytime incontinence) in that study [24]. There is evidence that radical treatment of MIBC even in geriatric patients is associated with a survival benefit. In an epidemiological study, 13.796 patients with MIBC were registered. For patients at an age ≥ 80 years, the greatest risk reduction in death from BC was shown for RC [13]. This underlines the results in our present study as we also could show RC to be associated with reduced OS and CSS in uni- and multivariable analyses in geriatric patients. The relatively short follow-up of 14 months in the present cohort should be reflected in the context of the study setting. Life expectancy per se is poor in an octogenarian cohort. Considering comorbidities and the presence of a severe urologic-oncological disease may explain the reduced life span and follow-up of the participants. Conservative treatment can be an alternative to radical treatment in geriatric patients with MIBC. In 68 patients, there was no difference in OS when patients were treated with RC (15.9 months) or conservatively (9.5 months). There was a trend towards better performance of patients treated with RC as we could also show in the present study [25].

Advanced age has been shown to represent a 2.2-fold increased risk for 90-day postoperative mortality [10]. To some extent, this is in line with the results of the present study as we demonstrate that geriatric patients are at a 7% risk for 30-days postoperative mortality regardless from the treatment procedure. In our opinion, this implicates the requirement of advanced interdisciplinary physical assessment before proceeding to plan any treatment options at all. Decision for any interventional treatment should consider this high risk for perioperative mortality during the first months.

In a study of 202 patients undergoing treatment for MIBC, the impact of patients` hemoglobin on OS and CSS was shown [26]. The results are in line with the present study as we also found low preoperative hemoglobin to be an independent risk factor for reduced OS and CSS. We checked our database for usage of anticoagulants and anti-platelets and found no correlation between drug use and low hemoglobin levels. Chronic tumor-related bleeding might be considered as one possible factor for low hemoglobin levels.

QoL in general was low throughout the whole cohort. Degboe et al. presented a study of 182 patients diagnosed with urothelial carcinoma of the upper and lower urinary tract. Baseline FACT-G score was 77.2 [27]. This score was considerably higher than the median baseline score in the present cohort which was assessed at 43.8 points. Younger patient`s age and better clinical performance throughout in the compared study may explain the differences in pretreatment QoL. In a study of 259 patients, Allareddy et al. showed a median FACT-G post treatment score of 89. RC was performed in 82 cases, in another 177 cases, a bladder preserving approach was done. In that study as well, the patients were younger (median age at diagnosis 64.4 years) than in the present cohort [28]. We think there are several potential reasons for similar results in Clavien–Dindo grade complications in the TURBT and the RC group in the present study. 54.9% of grade 2/3 complications in the RC group is in line with the results of other published RC cohorts in the literature reporting complications between 39 and 68% [29–31]. On the other hand, grade 2/3 complications for TURBT of 65.2% is seemingly higher compared to other studies reporting complication rates between 8.1 and 19.1% [32, 33]. However, these published cohorts were not selected for elderly patients and included a large number of non-muscle-invasive bladder carcinomas. Therefore, these studies are hardly comparable with our elderly MIBC patient group. In addition, in the study by Bansal et al., the complication rates of 24.5% after TURBT were significantly higher in elderly patients (> 60 years) compared to those of 17.5% in patients younger than 60 years. That study therefore reveals patients’ age as a risk factor of postoperative complications. In lack of studies in elderly MIBC patients with available reporting of complications, we can assume that the high complication rate in the TURBT group in the present collective is at least also related to the higher age of patients. Further prospective studies are needed to prospectively evaluate and specify the reasons of this phenomenon. We also can report about general statements from the patients who told us that repeated hospital admissions, endoscopic examinations and frequent medical checks were affecting QoL in the TURBT group. These physical and emotional stressors might explain the worse effect on FACT-G scoring in the EWB and PWB subgroups of the TURBT patients and might also explain the missing effect on SWB as social and family circumstances are less affected by the choice of a treatment procedure in the individual patient. The RC patients might suffer from an extended hospitalization time after surgery, but only some of the patients in the RC group experience an impairment of QoL by frequent invasive follow-up examinations. In contrast, all TURBT patients are intended for periodic invasive examinations (endoscopy and recurrent TURBT). This circumstance might explain why TURBT patients are “as dissatisfied” as RC patients and do not perform much better than the RC patients as we assumed before performing the present study.

We were surprised about the low QoL in our cohort. Taken together with a median survival time of 12 months, this result leaves us stirred as we feel having failed to improve the situation of our patients more sustainably. In our opinion, the “lesson learned” aspect of our results should be to further extend the interdisciplinary approach and geriatric assessment in the treatment of these critically ill patients and to investigate which future steps are necessary to improve treatment from the perspective of our patients. We are looking forward to include other treatment modalities, such as radiotherapy and most recently immunotherapy.

### Limitations

This study has some potential limitations due to its retrospective character. Comparability of the RC and the TURBT group is limited as histopathology examination of RC tissue resulted in upstaging of local tumor expansion or lymph node status in approximately half of the cases. For this purpose, in the present study, we compared the preoperative CT-scan-based clinical tumor staging in both groups. This is not as precise as comparison of histopathological specimen between two groups but helped to avoid comparison of histopathological results with radiological findings.

Decision-making on the individual treatment was not a randomized procedure in the present cohort. Recommendations of the institutional tumor board, a geriatric assessment and also patient`s choice were taken into account. This results in an inhomogeneous distribution of the patients in the different treatment groups. But regarding the small number of geriatric patients with MIBC at all, initiation of a prospective clinical trial to evaluate treatment options seems ambitious.

## Conclusion

RC and TURBT are feasible treatment options for MIBC in geriatric patients. Long-term survival is rare but possible, featuring both procedures. Since perioperative mortality is high in both treatment options, interdisciplinary and geriatric assessment should be considered before decision-making for any interventional treatment. CSS, OS and QoL were increased in the RC group.

## Data Availability

Data are available on reasonable request.
